# Cultural Adaptation and Psychometric Validation of the Standardised Nordic Questionnaire Spanish Version in Musicians

**DOI:** 10.3390/ijerph17020653

**Published:** 2020-01-19

**Authors:** Rosa Gómez-Rodríguez, Belén Díaz-Pulido, Carlos Gutiérrez-Ortega, Beatriz Sánchez-Sánchez, María Torres-Lacomba

**Affiliations:** 1 Physiotherapist in Private Clinic (IMA Fisiosalud), 28850 Madrid, Spain; rosagomrod@gmail.com; 2 Physiotherapy Department, Faculty of Medicine and Health Sciences, University of Alcalá, Alcalá de Henares, 28805 Madrid, Spain; belen.diazp@uah.es; 3 Medical Statistics Unit, Department of Epidemiology, Hospital Central de la Defensa, 28047 Madrid, Spain; cgutort@oc.mde.es; 4 Physiotherapy in Women’s Health Research Group, Physiotherapy Department, Faculty of Medicine and Health Sciences, University of Alcalá, Alcalá de Henares, 28805 Madrid, Spain; maria.torres@uah.es

**Keywords:** Standardised Nordic Questionnaire, reliability, validity, validation, musculoskeletal symptoms, Spanish, musicians

## Abstract

Background: The Standardised Nordic Questionnaire (SNQ) is an instrument to analyse the musculoskeletal symptoms in an ergonomic or occupational health context. We aimed to cross-culturally adapt and evaluate the psychometric properties of the SNQ among Spanish musicians. Methods: Cross-cultural adaptation and psychometric validation (reliability, validity, and feasibility) was performed. Reliability was analysed by test-retest reliability (Cohen’s Kappa) and internal consistency (Kuder–Richardson). Content and face validity were measured by the Expert Committee and the opinion of participants. Construct validity (Mann–Whitney U test) was measured by comparing with questionnaires used to assess pain and disability in neck, shoulders, upper back, and low back regions. Feasibility was calculated with the average response time. Results: A total of 312 Spanish musicians were included. The Spanish version of SNQ achieved good semantic, conceptual, idiomatic, and content equivalence. For most of the variables, test-retest reliability was good to very good (k = 0.60–0.81). The internal consistency showed good to acceptable (Kuder–Richardson 20 (KR20) = 0.737–0.873). Participants with versus without musculoskeletal problems in a related region showed significantly higher disability/pain, indicating a good construct validity. About the feasibility, the average response time of the questionnaire was 6 min (±2). Conclusions: The results show that the Spanish SNQ is reliable, valid, and feasible screening tool to assess musculoskeletal problems among musicians.

## 1. Introduction

Musicians require a long training, dedication, study, and practice, performing often into repetitive movements and maintaining postures for long time. These factors can cause pain and musculoskeletal disorders, in fact, playing-related musculoskeletal disorders are one of the main medical problems among musicians [1]. Pain is the primary symptom, but it may be variably described as aching, burning, electrical, or pulsating [2]. Reported point prevalence rates of musculoskeletal complaints varied from 57% to 68% for all musculoskeletal complaints, and from 9% to 68% for playing-related complaints. Playing-related 12-month prevalence ranged between 62% and 93% [3]. The neck, shoulders, and low back were the most frequently affected regions [3,4].

Thus, the evaluation of playing-related musculoskeletal disorder in the musician´s occupational context is an important outcome, in order to quantify, classify, and design an adequate treatment. The most commonly used instrument to detect the musician with symptoms is the Standardised Nordic Questionnaire (SNQ) [5,6,7,8,9].

The SNQ was developed in 1987 by Kuorinka et al. from a project funded by the Nordic Council of Ministers. It is an internationally self-administered questionnaire designed to evaluate musculoskeletal problems in an ergonomic or occupational health context. It consists in two parts: The general part and the specific lumbar, neck, and shoulders questionnaires. The SNQ has proved to be a valid, reliable, and feasible tool that allows for comparison of musculoskeletal problems among different anatomical areas in epidemiological studies [10]. With these studies, specific health promotion and prevention measures can be designed for each work environment. In general, it shows a good concordance with the functional clinical evaluation, but it should not be used as a tool to confirm the diagnosis of a disorder or a pathology, because it presents an important amount of false positives [11].

The SNQ has been adapted and validated to other languages [11,12,13,14,15,16,17], but the Spanish version has not been found. In order to analyse the situation of the musician in Spain, a cultural adaptation and validation of SNQ to the Spanish population is necessary. It will allow to compare results in Spain with those of other countries or between different labour population, and to draw conclusions about the relationship of these symptoms with other factors.

The aim of the present study has been adapting the original English SNQ into Spanish, and evaluating its psychometric properties of validity, reliability, and feasibility among Spanish musicians.

## 2. Materials and Methods

### 2.1. Study Design, Participants, and Procedure

A cross-sectional observational study was conducted from November 2016 to May 2018. The study reporting followed the “Strengthening the Reporting of Observational studies in Epidemiology” (STROBE) guidelines [18].

Participants were musicians recruited from public and private music schools, conservatories, and orchestras in the Community of Madrid (Spain), who fulfilled the inclusion criteria: They had played at least one musical instrument, for a minimum of 5 h per week, for over 16 years; and were native Spanish speakers, thus being able to read and to understand the Spanish language. An exponential discriminative snowball sampling technique was used. It was distributed a package of online open questionnaire by email, WhatsApp, and via Facebook, that included information about the study and the informed consent. Before starting the questionnaire, the participant had to express their consent by clicking on “NEXT”. Then, he had access to the sociodemographic and instrumental practice data, and to the Spanish versions of the Standardised Nordic Questionnaire, the Oswestry Disability Index, the Neck Disability Index, and the Shoulder Pain and Disability Index.

The package of questionnaires was administrated by means of the Google Forms platform, which allows to access and to reply the instrument from any type of electronic device with an Internet connection. Validations of questionnaires have already been done using online applications, proving them to be a useful tool [19,20].

The response rate could not be controlled.

### 2.2. Translation and Cultural Adaptation

The study was developed in three phases according to the International Society for Pharmacoeconomics and Outcomes Research (ISPOR) [21].

#### 2.2.1. Cultural Translation and Adaptation

Firstly, the original version of SNQ was translated into Spanish by two English–Spanish translators, who (native Spanish speakers) worked independently. The translators and the research team agreed the Spanish translation synthesis. Then, this first Spanish version was back-translated into English by two bilingual Spanis–English translators (native English speakers) worked independently from one another, to verify that the translation reflected the same content as the original. With the first version of the questionnaire and with the back-translated versions, an Expert Committee agreed the preliminary SNQ Spanish version was equivalent to the original instrument.

#### 2.2.2. Pilot Test

The preliminary SNQ Spanish version was administered to 25 participants who fulfilled the inclusion criteria to reach the final SNQ version. They self-completed the questionnaire, and afterwards, they were interviewed in order to identify and correct potential understanding difficulties of the items and the quality of cultural adjustment. Finally, SNQ Spanish version was obtained.

#### 2.2.3. Psychometric Validation

The SNQ Spanish version was administered until it reached a sample of at least 136 symptomatic participants and 80 asymptomatic participants. The sample size has been based on the general recommendations of Altman [22] and Terwee et al. [23], that recommend at least 50 subjects for the evaluation of the measures; and on Bryant & Yarnold [24], who recommend that the relationship between participants and variables be not less than five [25]. The sociodemographic data of the entire sample were collected. To perform the analysis of convergent construct validity, participants were asked to fill in, in addition to the SNQ Spanish version, the Oswestry Disability Index [26], the Neck Disability Index [27], and Shoulder Pain and Disability Index [28].

### 2.3. Instruments

#### 2.3.1. The Standardised Nordic Questionnaire (SNQ)

The SNQ is divided in two parts, the general and the specific part. The general part of the SNQ consists of 27 questions with dichotomous response (Yes/No) about musculoskeletal symptoms during the last 12 months or the last 7 days and about the impact on activities during the last 12 months [10]. All these questions refer to 9 areas: Neck, shoulders, elbows, wrists/hands, upper back, low back, hips/thighs, knees and ankles/feet. For facilitating the identification of the anatomical areas, it also includes a corporal diagram seen from behind. The specific parts of the questionnaire delve into the analysis of symptoms of the lumbar, neck and shoulder regions with dichotomous response (Yes/No) or with the timing of the problem [10].

#### 2.3.2. The Oswestry Disability Index

The Oswestry Disability Index is a self-applied questionnaire specific for low back pain that measures limitations in daily activities that can be affected by pain and is validated in Spanish [29]. Higher scores show a higher level of disability [26].

#### 2.3.3. The Neck Disability Index

The Neck Disability Index is a self-completed questionnaire specific for cervical pain that measures limitations in daily activities. It is validated in Spanish [30]. A higher score shows a higher level of disability [27].

#### 2.3.4. The Shoulder Pain and Disability Index

The Shoulder Pain and Disability Index is a self-reporting questionnaire that measures shoulder pain and disability. It is validated in Spanish [31]. Higher scores in each subscale imply greater pain intensity and greater disability [28].

### 2.4. Psychometric Validation Process

The SNQ Spanish version was tested for reliability, validity, and feasibility.

Reliability was assessed by internal consistency and test-retest reliability. The internal consistency, which is determined by the degree to which all elements measure the same, has been studied by comparing the answers to the questions about the troubles and/or disability in neck, shoulder, or low back regions contained in the general questionnaire and the dichotomous questions contained in the corresponding specific questionnaires. It was measured using Kuder–Richardson formula (KR20), which ranges from 0 to 1. Values above 0.9 are considered, excellent; between 0.8 and 0.89, good; between 0.7 and 0.79, acceptable; between 0.6 and 0.69, questionable; and below 0.6, poor or unacceptable [32]. Test-retest reliability is the degree to which a measuring instrument provides stable results on repeated administration when the domains to be measured have not changed [33]. It was assessed in 25 participants who completed the questionnaire a second time, 3–4 days after the first test, and it was evaluated by the Cohen´s Kappa coefficient (k), for which the values range from 0 (without agreement) to 1 (perfect agreement). Values above 0.81 show very good agreement; between 0.61 and 0.8, good; between 0.41 and 0.6, moderate; between 0.21 and 0.4, weak, and below 0.20, poor [22].

Validity identifies the degree to which an instrument measures what it is designed for. This was assessed through content, face, and construct validity. The content validity was evaluated by the Expert Committee created for the translation and cultural adaptation of the questionnaire to the Spanish version. Face validity was evaluated by the opinion of the participants in the pilot study, who analysed the scale and decided whether it really seemed to measure what it was proposed for. Construct validity was measured by comparison with questionnaires that are used to assess pain and disability in neck, shoulder, upper back, and low back regions. Answers of SNQ were dichotomous, since we could not measure the construct validity by using correlation analysis. Therefore, we hypothesised that participants with musculoskeletal problems in a related region would have significantly higher disability/pain levels as assessed by the relevant questionnaires. Because these instruments are relevant to short-term situations, it was analysed using items regarding musculoskeletal symptoms during the last 7 days of the general questionnaire of the SNQ. This construct validity was evaluated using the Mann–Whitney U test [16]. For that, the result correlations of the SNQ Spanish version with the Oswestry Disability Index, the Neck Disability Index and the Shoulder Pain and Disability Index Spanish versions were calculated.

To evaluate feasibility, the average administration time was calculated by the mean.

A *p* value < 0.05 was considered statistically significant.

The data were performed using the IBM ^®^ Statistical Package SPSS, version 24.0 (IBM, Armonk, NY, USA).

## 3. Results

### 3.1. Translation and Cultural Adaptation

Translation and cultural adaptation of the questionnaire revealed no difficulties. Only the question regarding work experience time was modified, asking the subject only for the years of experience instead of for the months and years, facilitating the fill in of the questionnaire. After analysing the records for participants’ doubts and suggestions, consensus about translation of the SNQ was obtained and a definitive questionnaire was conducted. The cross-cultural adaptation of the SNQ Spanish version achieved a good semantic, conceptual and content equivalence (Supplementary Material Table S1).

### 3.2. Psychometric Validation Process

A total of 361 responses were received. Forty-nine responses have been excluded for not meeting the inclusion criteria and 312 responses have been included. Of the total sample, 160 (51.3%) musicians were men. The median age was 25 with an interquartile range (IQR) of 16. The average body mass index was 23.43 with a standard deviation of 3.89. The median number of years playing was 15 (IQR 13.75) and the median hours of weekly practice was 10 (IQR 14). Regarding the instrumental group, 97 musicians (33.1%) played string, 35 (11.9%) keyboard, 99 (33.8%) woodwind, 34 (11.6%) brass, and 28 (9.6%) percussion.

#### 3.2.1. Test-Retest Reliability

In the analysis of the test-retest reliability of the general questionnaire, only the question regarding the dorsal area and the prevention from doing normal work at home or away from home showed a weak agreement (k = 0.359 (95% CI: -)); for the lumbar area, a moderate agreement was obtained (k = 0.595 (95% CI: 0.183–1.000)). For the other variables in the general questionnaire, good and very good agreements were obtained. For some variables, it was not possible to compute the test, because all individuals gave the same answer for the two applications of the questionnaire. The Kappa agreement correlation coefficient of the general questionnaire can be seen in Table 1.

In the specific questionnaires, the variable related to the reduction of leisure activities during the last 12 months due to neck problems obtained a weak agreement (k = 0.254 (95% CI: -)) and the variables related to having neck problems at some point in life and if lumbar problems had caused to change jobs or duties obtained a moderate agreement (k = 0.503 (95% CI: 0.019–0.988)). The rest of the variables obtained good and very good results. The results are shown in Table 2.

#### 3.2.2. Internal Consistency

The internal consistency of all the variables of each region was good (neck, KR20 = 0.817 (95% CI: 0.786–0.846); shoulders, KR20 = 0.873 (95% CI: 0.851–0.893); lumbar, KR20 = 0.839 (95% CI: 0.811–0.865)). For the severity of the problem in the shoulders, a good internal consistency was also obtained (KR20 = 0.856 (95% CI: 0.830-0.879)). For the rest, acceptable values were obtained, as available in Table 3. The internal consistency of the general questionnaire was 0.835 (95% CI: 0.807–0.860).

#### 3.2.3. Construct Validity

In the evaluation of construct validity, there was a significant difference in the neck and shoulder disability/pain level assessed respectively by the Neck Disability Index and the Shoulder Pain and Disability Index, among participants with musculoskeletal problems in the neck or shoulder region versus participants without them. The participants who reported upper and low back problems had significantly more disability assessed by Oswestry Disability Index. The construct validity results are showed in Table 4.

#### 3.2.4. Average Response Time

The average response time of the questionnaire was 6 min (±2).

## 4. Discussion

The SNQ is a questionnaire that allows for examining the extent of a problem and recognizing its importance in the workplace. It is a first step to see if there are musculoskeletal health problems and to evaluate the evolution of the situation, although it does not allow for attributing the causes of the problems [34]. It is a widely used questionnaire because it is easy and quick to answer. It is validated in Turkish [16], European Portuguese [12], Brazilian Portuguese [13], Greek [15], Chinese [17], Italian [14], and Chilean [11].

The translation/back translation process used to obtain SNQ’s Spanish version has been similar to other SNQ validations [12,13,14,15,16]. The linguistic adaptation process showed that musicians easily understand SNQ’s Spanish version. At the end of the questionnaire, a section for comments was added in, and several musicians suggested asking about musculoskeletal problems in the face area, due to the orofacial problems presented by musicians.

In the present study, the sample size was 312 musicians. The sample calculation indicated that at least 136 responses were necessary. The Turkish validation calculates the sample size using the internal consistency data of the European Portuguese and estimating an interval confidence of 95%. In total, they estimated a sample size of 193 participants [16]. The Chilean validation calculated the sample size based on the method used in the original validation [10]. They deemed it necessary to include 20 participants by employment title, so to assess six different work-types, they calculated the sample size in 120 participants [11]. The other validations do not estimate the sample size calculation. They used samples of 40 [13,14] or 60 participants [12]. The response rate could not be controlled.

The results of the test-retest reliability of the general questionnaire are good and very good for the specific questionnaires, good and very good values were also obtained. Four questions obtained a moderate agreement and one question obtained a weak agreement (two questions in general questionnaire and three questions in specific questionnaires). These questions refer to problems over the last 12 months or at some point in life, so memory bias may influence the responses of the participants. Moreover, except for the question referring to the presence of problems in the neck, which refers to the severity of the problem, the other questions with lower agreements refer to the impact on the activities. Between questions with good and very good agreements, this fact is also repeated, and better results are obtained for questions about the severity of the problem. A possible explanation is that for the participant it is easier to assess if he has had a problem and the intensity of it, than to assess the impact that this problem has actually caused on the activities. The other SNQ versions also obtained good and very good results for most of the questions [12,13,14,15,16]. In the Brazilian Portuguese version, they obtained a moderate agreement value for pain in the last seven days at the elbow. They think that, regardless of the stability of the instrument, the medical condition of the participant may change at the time of retesting. In addition, by answering a second time, they can respond in a careless manner, resulting in lower stability [13]. The lowest Kappa values were in a range between 0.21–0.40, that reflects weak agreement rates in these questions, but in any case, indicated no agreement or slight agreement. Therefore, no questions were excluded from the validated version and it has been decided to keep the same questions as in the original version.

The agreement values have been analysed for questions that are repeated identically or similarly between the general questionnaire and the specific questionnaires. The questions about problems in the last seven days in the lumbar region and the neck, and about problems in the shoulder during the last 12 months, have obtained equal or similar values, while for the variable shoulder problems in the last seven days, a moderate agreement in the general questionnaire was obtained (k = 0.615 (95% CI = 0.296–0.934)), whereas in the specific questionnaire, a perfect agreement value was obtained (k = 1 (95% CI = -)). One explanation for this could be that better agreements are obtained in the specific questionnaires, but they are slightly higher in the other variables of the general questionnaire.

In the present study, the internal consistency has been calculated with the KR20 formula. This is a specific case of Cronbach’s alpha for dichotomous questions. The internal consistency of the general questionnaire is good (KR20 = 0.835), similar to the Turkish validations (Cronbach’s alpha = 0.896) [16] and the European Portuguese (KR20 = 0.855) [12]. However, we believe that these values can be the result of chance, since we would have to assume a correlation between variables, such as having problems in the ankle and having problems in the neck, body areas that are not necessarily related. For this reason, similar to the work done in the Italian validation, we compared the questions of the body areas lumbar, neck, and shoulder of the general questionnaire with their respective specific questionnaires, obtaining good values [14]. The internal consistency of the questions referred to the severity of the problem and the questions related to the impact on the activities have also been analysed separately, in a similar way to the online, extended version of the SNQ [19], finding good and acceptable values, being these values for the shoulder region, and for the items on the severity of the problem slightly higher.

A correlation analysis could not be used to analyse the construct validity. It was analysed in the same way as in the Turkish version. They hypothesised that participants with a musculoskeletal problem at related regions would have significantly more disability/pain measured with other relevant questionnaires [16]. Construct validity has been shown to be statistically significant for the assessed regions (lumbar, dorsal, neck and shoulders). One limitation of this study is not having analysed the rest of the body areas (lower limbs, elbows, and wrists/hands).

The average response time of the questionnaire was assessed in the test time and it was 6 min (±2). Other SNQ validations have not assessed this aspect of the questionnaire, so our result cannot be compared.

## 5. Conclusions

The results of the present study showed that the SNQ Spanish version has a semantic, conceptual, idiomatic, and content equivalence with the original version. It is an easy to apply questionnaire, as well as being a reliable, valid, and feasible screening tool to assess possible musculoskeletal problems within the workplace.

## Figures and Tables

**Table 1:** The Kappa agreement correlation coefficient for each answer in the general questionnaire (*n* = 25).

Location of the Trouble	Have You at Any Time During the Last 12 Months Had Trouble (Ache, Pain, Discomfort) in?		Have You at Any Time During the last 12 Months Been Prevented from Doing Your Normal Work (at Home or Away from Home) Because of the Trouble?		Have You Had Trouble at Any Time during the Last 7 Days?	
	Kappa	95% CI	Kappa	95% CI	Kappa	95% CI
Neck	0.752	0.434–1.000	0.694	0.384–1.000	0.763	0.519–1.000
Shoulders	1	-	0.651	0.290–1.000	0.615	0.296–0.934
Elbows	0.865	0.608–1.000	1	-	0.627	0.167–1.000
Wrists/hands	0.733	0.542–1.000	0.750	0.422–1.000	0.779	0.359–1.000
Upper Back	0.667	0.368–0.965	0.359	-	0.884	0.662–1.000
Low back	0.818	0.579–1.000	0.595	0.183–1.000	0.746	0.477–1.000
Hips/thighs	0.896	0.698–1.000	0 *	-	0.706	0.332–1.000
Knees	0.752	0.434–1.000	0 *	-	1	-
Ankles/feet	0.603	0.216–0.990	0 *	-	1	-

* Could not be computed because all variables were constant. CI: Confidence interval.

**Table 2:** The Kappa agreement correlation coefficient for each answer in the specific questionnaires (*n* = 25).

Low back			Neck			Shoulders		
Question	Kappa	95% CI	Question	Kappa	95% CI	Question	Kappa	95% CI
LBSQ1	1	-	NSQ1	0.503	0.019–0.988	SSQ9	0.689	0.364–1.000
LBSQ2	0.779	0.359–1.000	NSQ2	0.802	0.539–1.000	SSQ10	0.884	0.662–1.000
LBSQ3	0.503	0.019–0.988	NSQ3	0.834	0.521–1.000	SSQ11	1	-
LBSQ5a	0.621	0.141–1.000	NSQ5a	0.884	0.662–1.000	SSQ12	0.841	0.632–1.000
LBSQ5b	0.834	0.521–1.000	NSQ5b	0.254	-	SSQ14a	0.896	0.698–1.000
LBSQ7	1	-	NSQ7	0.920	0.766–1.000	SSQ14b	1	-
LBSQ8	0.746	0.477–1.000	NSQ8	0.688	0.421–0.954	SSQ16	0.834	0.617–1.000
						SSQ17	1	-

NSQ: Neck specific questionnaire; SSQ: Shoulder specific questionnaire; LBSQ: Low back specific questionnaire. CI: confidence interval.

**Table 3:** Internal consistency verified by the Kuder–Richarson coefficient of reliability (*n* = 312).

Construct	Subscale	Items	KR20	95% CI
Musculoskeletal trouble in neck region	All items in neck	NSQ (1,2,3,5a,5b,7,8) + GQN (1,2,3)	0.817	0.786–0.846
	Severity of symptoms	NSQ (1,2,7,8) + GQN (1,3)	0.774	0.732–0.811
	Impact on activities	NSQ (3,5a,5b) + GQN 2	0.737	0.686–0.782
Musculoskeletal trouble in shoulder region	All items in shoulder	SSQ (9,10,11,12,14a,14b,16,17) + GQS (1,2,3)	0.873	0.851–0.893
	Severity of symptoms	SSQ (9,10,12,16,17) + GQS (1,3)	0.856	0.830–0.879
	Impact on activities	SSQ (11,14a,14b) + GQS 2	0.783	0.741–0.820
Musculoskeletal trouble in low back region	All items in low back	LBSQ (1,2,3,5a,5b,7,8) + GQLB (1,2,3)	0.839	0.811–0.865
	Severity of symptoms	LBSQ (1,2,7,8) + GQLB (1,3)	0.789	0.751–0.824
	Impact on activities	LBSQ (3,5a,5b) + GQLB 2	0.749	0.700–0.792

KR20 of the general questionnaire = 0.835 (95% CI: 0.807-0.860).KR: Kuder–Richarson; NSQ: Neck specific questionnaire; GQN: General questionnaire, neck; SSQ: Shoulder specific questionnaire; GQS: General questionnaire, shoulders; LBSQ: Low back specific questionnaire; GQLB: General questionnaire, low back. CI: confidence interval.

**Table 4:** Comparison of the disability levels assessed by the relevant questionnaires between the participants with versus without a musculoskeletal problem during the last 7 days (*n* = 312).

Musculoskeletal Problem during the Last 7 Days	Yes		No		Mann–Whitney U Test	*p* Value
	Median	IQR	Median	IQR		
Neck (a)	14.0	24.0–6.0	2.0	8.0–0.0	4279.500	<0.001
Shoulders (b)	22.31	38.46–10.0	3.85	16.54–0.0	5450.000	<0.001
Upper back (c)	8.0	17.0–4.0	4.0	10.0–0.0	5847.500	<0.001
Low back (c)	8.0	16.0–4.0	4.0	8.0–0.0	6134.500	<0.001

Md: Median; IQR: Interquartile range; (a) Neck disability index; (b) Shoulder pain and disability index; (c) Oswestry low back disability index.

## Supplementary Materials

The following are available online at https://www.mdpi.com/1660-4601/17/2/653/s1, Table S1: The SNQ Spanish Version.
