# Supplementary material for: Cultural Adaptation and Psychometric Validation of the Standardised Nordic Questionnaire Spanish Version in Musicians
Source: Int J Environ Res Public Health. 2020 Jan 19;17(2):653. doi: 10.3390/ijerph17020653 (PMC7014211; doi:10.3390/ijerph17020653)
Supplement: Supplementary file 1 [file ijerph-17-00653-s001.pdf]

CUESTIONARIO NÓRDICO ESTÁNDAR

Fecha de la investigación ___ / ___ / _____
Sexo:

1 □ Mujer
2 □ Hombre

¿En qué año ha nacido? ______

¿Cuánto pesa? ____ kg.

¿Cuánto mide? ____ cm.

¿Cuántos años y meses lleva tocando un instrumento? ___ años + ___ meses

¿Cuántas horas de media toca a la semana? ____ horas/semana

¿Es zurdo o diestro?

1 □ Diestro
2 □ Zurdo

Cómo contestar al cuestionario: Por favor, conteste marcando con una cruz la casilla apropiada (una cruz por pregunta). Si tiene dudas sobre cómo responder a alguna cuestión, pregunte al investigador. Conteste a cada una de las preguntas, incluso si no ha tenido problemas en alguna de las zonas de su cuerpo.

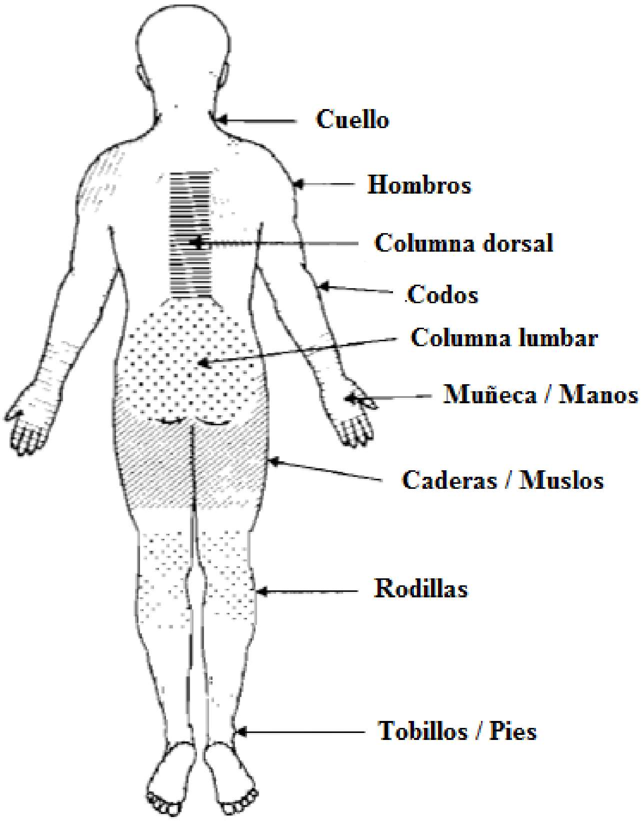

En esta imagen puede ver la localización aproximada de las zonas del cuerpo a las que se refiere el cuestionario. Los límites no están claramente definidos y ciertas zonas están superpuestas. Debe decidir usted mismo en qué zona tiene o ha tenido su problema.

Problemas en el aparato locomotor		
Ha tenido en los últimos 12 meses algún problema (incomodidad, malestar o dolor) en	Conteste sólo si ha tenido problemas	
	¿Ha tenido **durante los últimos 12 meses algún momento en que no haya podido hacer su trabajo normal** (en casa o fuera de ella) a causa del problema?	¿Ha tenido algún problema **durante los últimos 7 días**?
**Cuello**<br>1 □ No 2 □ Sí	1 □ No 2 □ Sí	1 □ No 2 □ Sí
**Hombros**<br>1 □ No 2 □ Sí, en el hombro derecho<br>3 □ Sí, en el hombro izquierdo<br>4 □ Sí, en ambos hombros	1 □ No 2 □ Sí	1 □ No 2 □ Sí
**Codos**<br>1 □ No 2 □ Sí, en el codo derecho<br>3 □ Sí, en el codo izquierdo<br>4 □ Sí, en ambos codos	1 □ No 2 □ Sí	1 □ No 2 □ Sí
**Muñecas / Manos**<br>1 □ No 2 □ Sí, en la muñeca / mano derecha<br>3 □ Sí, en la muñeca / mano izquierda<br>4 □ Sí, en ambas muñecas / manos	1 □ No 2 □ Sí	1 □ No 2 □ Sí
**Columna dorsal**<br>1 □ No 2 □ Sí	1 □ No 2 □ Sí	1 □ No 2 □ Sí
**Columna lumbar**<br>1 □ No 2 □ Sí	1 □ No 2 □ Sí	1 □ No 2 □ Sí
**Una o ambas caderas / muslos**<br>1 □ No 2 □ Sí	1 □ No 2 □ Sí	1 □ No 2 □ Sí
**Una o ambas rodillas**<br>1 □ No 2 □ Sí	1 □ No 2 □ Sí	1 □ No 2 □ Sí
**Uno o ambos tobillos / pies**<br>1 □ No 2 □ Sí	1 □ No 2 □ Sí	1 □ No 2 □ Sí

Columna lumbar

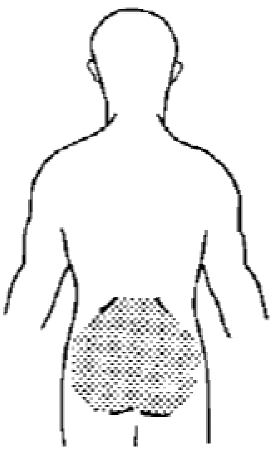

Cómo contestar al cuestionario: En esta imagen puede ver la localización aproximada de la zona a la que se refiere el cuestionario. Por problemas de la columna lumbar se entiende incomodidad, malestar o dolor en el área sombreada, extendido o no desde allí hasta una o ambas piernas (ciática).

Por favor, conteste marcando una cruz en la casilla apropiada (una cruz por pregunta). Si tiene dudas sobre cómo responder a alguna cuestión, pregunte al investigador. Conteste a cada pregunta, incluso si no ha tenido problemas en alguna de las zonas de su cuerpo.

1 ¿Alguna vez ha tenido problemas (incomodidad, dolor o malestar) en la región lumbar?
1 □ No 2 □ Sí

Si contesta **NO a la pregunta 1**, no conteste a las preguntas 2-8

2 ¿Alguna vez ha sido hospitalizado por problemas en la región lumbar?
1 □ No 2 □ Sí

3 ¿Alguna vez ha cambiado trabajos u ocupaciones por problemas en la región lumbar?
1 □ No 2 □ Sí

4 ¿Cuánto tiempo en total ha tenido problemas en la región lumbar durante los últimos 12 meses?
1□ 0 días
2 □ 1-7 días
3 □ 8-30 días
4 □ Más de 30 días, pero no todos los días
5 □ Todos los días

Si ha contestado **0 días en la pregunta 4, no conteste a las preguntas 5-8**

5 ¿Su problema en la región lumbar le ha hecho reducir su actividad durante los últimos 12 meses?
a. Su actividad laboral (en casa o fuera de casa)
1 □ No 2 □ Sí
b. Su actividad de ocio
1 □ No 2 □ Sí

6 ¿Cuánto tiempo en total su problema de espalda le ha impedido hacer su trabajo normal (en casa o fuera de casa) durante los últimos 12 meses?
1 □ 0 días
2 □ 1-7 días
3 □ 8-30 días
4 □ Más de 30 días

7 ¿Le ha visto algún médico, fisioterapeuta, quiropráctico u otro profesional debido a problemas en la región lumbar durante los últimos 12 meses?
1 □ No 2 □ Sí

8 ¿Ha tenido problemas en la región lumbar durante los últimos 7 días?
1 □ No 2 □ Sí

Cuello

Cómo contestar al cuestionario: Por problemas de cuello se entiende incomodidad, malestar o dolor en el área sombreada. Por favor, concéntrese en esta área, ignorando cualquier problema que tenga en otras zonas adyacentes del cuerpo. Hay un cuestionario independiente/específico para los problemas de hombro.

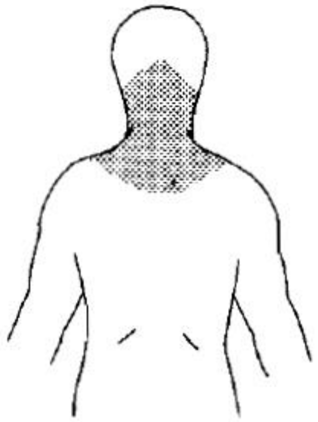

Conteste marcando una cruz en la casilla apropiada (una cruz por pregunta). Si tiene dudas sobre cómo responder a alguna cuestión, pregunte al investigador. Por favor, conteste a cada pregunta, incluso si no ha tenido problemas en alguna de las zonas del cuerpo.

1 ¿Alguna vez ha tenido problemas en el **cuello (incomodidad, malestar o dolor)?**<br>1 □ No 2 □ Sí

Si contesta **NO a la pregunta 1**, no conteste a las preguntas 2-8.

**2 ¿Alguna vez se ha dañado el cuello en algún accidente?**<br>1 □ No 2 □ Sí
**3 ¿Alguna vez ha cambiado trabajos u ocupaciones por problemas en el cuello?**<br>1 □ No 2 □ Sí
**4 ¿Cuánto tiempo en total ha tenido problemas en el cuello durante los últimos 12 meses?**<br>1 □ 0 días<br>2 □ 1-7 días<br>3 □ 8-30 días<br>4 □ Más de 30 días, pero no todos los días<br>5 □ Todos los días

Si ha contestado **0 días en la pregunta 4**, no conteste a las preguntas 5-8

**5 ¿Su problema en el cuello le ha hecho reducir su actividad durante los últimos 12 meses?**<br>a. Su actividad laboral (en casa o fuera de casa)<br>1 □ No 2 □ Sí<br>b. Su actividad de ocio<br>1 □ No 2 □ Sí
**6 ¿Cuánto tiempo en total su problema en el cuello le ha impedido hacer su trabajo normal (en casa o fuera de casa) durante los últimos 12 meses?**<br>1 □ 0 días<br>2 □ 1-7 días<br>3 □ 8-30 días<br>4 □ Más de 30 días
**7 ¿Le ha visto algún médico, fisioterapeuta, quiropráctico u otro profesional debido a problemas en el cuello durante los últimos 12 meses?**<br>1 □ No 2 □ Sí
**8 ¿Ha tenido problemas en el cuello durante los últimos 7 días?**<br>1 □ No 2 □ Sí

Hombros

Cómo contestar al cuestionario: Por problemas en el hombro se entiende incomodidad, malestar o dolor en el área sombreada. Por favor, concéntrese en esta área, ignorando cualquier problema que tenga en zonas adyacentes del cuerpo. Hay un cuestionario independiente/específico para los problemas de cuello.

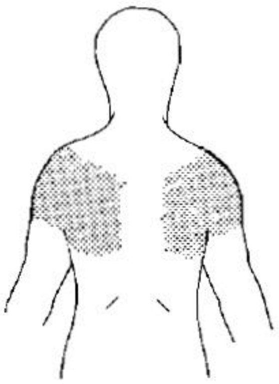

Conteste marcando una cruz en la casilla apropiada (una cruz por pregunta). Si tiene dudas sobre cómo responder a alguna cuestión, pregunte al investigador. Por favor, conteste a cada pregunta, incluso si no ha tenido problemas en alguna zona del cuerpo.

9 ¿Alguna vez ha tenido problemas en el hombro (incomodidad, malestar o dolor)?
1 □ No 2 □ Sí

Si contesta **NO a la pregunta 9**, no conteste a las preguntas 10-17.

10 ¿Alguna vez se ha hecho daño en el hombro en algún accidente?
1 □ No 2 □ Sí, en el hombro derecho
3 □ Sí, en el hombro izquierdo
4 □ Sí, en ambos hombros

11 ¿Alguna vez ha cambiado trabajos u ocupaciones por problemas en el hombro?
1 □ No 2 □ Sí

12 ¿Ha tenido problemas en el hombro durante los últimos 12 meses?
1 □ No 2 □ Sí, en el hombro derecho
3 □ Sí, en el hombro izquierdo
4 □ Sí, en ambos hombros

Si ha contestado **NO en la pregunta 12**, no conteste a las preguntas 13-17

13 ¿Cuánto tiempo en total ha tenido problemas en el hombro durante los últimos 12 meses?
1 □ 0 días
2 □ 1-7 días
3 □ 8-30 días
4 □ Más de 30 días, pero no todos los días
5 □ Todos los días

14 ¿Su problema en el hombro le ha hecho reducir su actividad durante los últimos 12 meses?
a. Su actividad laboral (en casa o fuera de casa)
1 □ No 2 □ Sí
b. Su actividad de ocio
1 □ No 2 □ Sí

15 ¿Cuánto tiempo en total su problema en el hombro le ha impedido hacer su trabajo normal (en casa o fuera de casa) durante los últimos 12 meses?
1 □ 0 días
2 □ 1-7 días
3 □ 8-30 días
4 □ Más de 30 días

16 ¿Le ha visto algún médico, fisioterapeuta, quiropráctico u otro profesional a causa de problemas en el hombro durante los últimos 12 meses?
1 □ No 2 □ Sí

17 ¿Ha tenido problemas en el hombro durante los últimos 7 días?
1 □ No 2 □ Sí
